# Supplementary material for: MetaRibo-Seq measures translation in microbiomes
Source: Nat Commun. 2020 Jun 29;11:3268. doi: 10.1038/s41467-020-17081-z (PMC7324362; doi:10.1038/s41467-020-17081-z)
Supplement: Supplementary file 10 — Supplementary Data 7 [file 41467_2020_17081_MOESM10_ESM.zip › File2/Confidence_High_Taxonomy/386438_out.krona.html]

Javascript must be enabled to view this page.

members
magnitude
magnitudeUnassigned
count
unassigned
taxon
rank

386438\_out

391


SRS054352\_contig\_number\_contig-100\_24512.62808
391
superkingdom
2
1

976
phylum
390

class
200643
390

390
order
171549

171552
family
1

838
genus
1

59823
species
1

SRS019161\_contig\_number\_3907

family
815
384


SRS012849\_contig\_number\_contig-100\_3067.214129SRS013638\_contig\_number\_contig-100\_3741.103741SRS013687\_contig\_number\_15855SRS014459\_contig\_number\_3958SRS015190\_contig\_number\_contig-100\_2460.100374SRS015579\_contig\_number\_contig-100\_3290.138615SRS015782\_contig\_number\_contig-100\_4994.219462SRS015854\_contig\_number\_contig-100\_1916.128360SRS016381\_contig\_number\_353SRS017701\_contig\_number\_11007SRS017821\_contig\_number\_884SRS017916\_contig\_number\_9654SRS019030\_contig\_number\_11564SRS022137\_contig\_number\_18189SRS022524\_contig\_number\_contig-100\_2022.76279SRS023583\_contig\_number\_11187SRS023914\_contig\_number\_contig-100\_1706.150304SRS023971\_contig\_number\_822SRS042628\_contig\_number\_32324SRS042628\_contig\_number\_contig-100\_2551.192577SRS047014\_contig\_number\_22212SRS049446\_contig\_number\_10555SRS050520\_contig\_number\_contig-100\_1298.1299SRS050974\_contig\_number\_contig-100\_5326.5327SRS050998\_contig\_number\_contig-100\_1048.109129SRS051882\_contig\_number\_contig-100\_4130.128032SRS052027\_contig\_number\_18297SRS055982\_contig\_number\_6188SRS056273\_contig\_number\_contig-100\_2596.155667SRS057480\_contig\_number\_contig-100\_9397.55100SRS058145\_contig\_number\_contig-100\_6952.6953SRS063040\_contig\_number\_contig-100\_11015.80252SRS063489\_contig\_number\_contig-100\_880.36534SRS074586\_contig\_number\_contig-100\_5446.46219SRS075078\_contig\_number\_25430SRS077454\_contig\_number\_18502SRS097920\_contig\_number\_3176SRS100021\_contig\_number\_11394SRS100907\_contig\_number\_7441SRS101448\_contig\_number\_contig-100\_1870.1870SRS1041141\_contig\_number\_contig-100\_2001.60953SRS1041143\_contig\_number\_8982SRS1055056\_contig\_number\_contig-100\_4362.81562SRS1055095\_contig\_number\_2362SRS142781\_contig\_number\_contig-100\_1563.88691SRS142890\_contig\_number\_32555SRS143722\_contig\_number\_15031SRS143876\_contig\_number\_31775SRS144753\_contig\_number\_6502SRS147088\_contig\_number\_contig-100\_6377.6377SRS147271\_contig\_number\_40321SRS148196\_contig\_number\_20968SRS148253\_contig\_number\_8925SRS149181\_contig\_number\_31049SRS893256\_contig\_number\_1548SRS893295\_contig\_number\_4612SRS893327\_contig\_number\_contig-100\_4306.71504SRS893342\_contig\_number\_3542
58
genus
816
384


SRS018541\_contig\_number\_3529SRS101268\_contig\_number\_838
2
2292927
species


SRS016335\_contig\_number\_11930SRS104693\_contig\_number\_11767
2
species
338188

1
469585
species

SRS016203\_contig\_number\_8605


SRS015890\_contig\_number\_460SRS022713\_contig\_number\_1570SRS046068\_contig\_number\_1015SRS048870\_contig\_number\_8886SRS1055043\_contig\_number\_14677
5
818
species


SRS011405\_contig\_number\_14213SRS056259\_contig\_number\_12503SRS104636\_contig\_number\_14015SRS142599\_contig\_number\_15657
species
329854
4


SRS013951\_contig\_number\_36972SRS014979\_contig\_number\_16586SRS016840\_contig\_number\_2246SRS021948\_contig\_number\_2128SRS065504\_contig\_number\_2299SRS1041039\_contig\_number\_27473SRS1041137\_contig\_number\_15540SRS144183\_contig\_number\_5521SRS892882\_contig\_number\_4839SRS893373\_contig\_number\_24547
species
2292284
10


SRS013638\_contig\_number\_11950SRS019601\_contig\_number\_12831SRS057478\_contig\_number\_1721SRS1041130\_contig\_number\_667SRS1055083\_contig\_number\_467SRS147346\_contig\_number\_12633SRS147653\_contig\_number\_7287SRS893252\_contig\_number\_1538SRS893253\_contig\_number\_992
species
821
9


SRS047433\_contig\_number\_12793
671267
species
1


SRS011084\_contig\_number\_4202SRS020622\_contig\_number\_417SRS024331\_contig\_number\_15720SRS078419\_contig\_number\_33145
4
28116
species

1
species
93975

SRS055017\_contig\_number\_23777


SRS011239\_contig\_number\_9239SRS013098\_contig\_number\_37352SRS015431\_contig\_number\_38231SRS016495\_contig\_number\_18535SRS019445\_contig\_number\_26120SRS019685\_contig\_number\_20112SRS024492\_contig\_number\_contig-100\_161.74492SRS043411\_contig\_number\_11850SRS052697\_contig\_number\_33456SRS053214\_contig\_number\_14777SRS062701\_contig\_number\_5754SRS075773\_contig\_number\_3797SRS097889\_contig\_number\_14558SRS097958\_contig\_number\_9976SRS142923\_contig\_number\_19623SRS143148\_contig\_number\_18553SRS148784\_contig\_number\_2048SRS892882\_contig\_number\_9029
246787
species
18


SRS017521\_contig\_number\_19339SRS019267\_contig\_number\_5892SRS049712\_contig\_number\_contig-100\_1117.108999SRS050299\_contig\_number\_1084SRS075878\_contig\_number\_848SRS893342\_contig\_number\_1429
6
species
817

1262754
species
1

SRS043768\_contig\_number\_4618

species
820
145

SRS011239\_contig\_number\_22402SRS011302\_contig\_number\_22271SRS012969\_contig\_number\_contig-100\_1176.218177SRS013521\_contig\_number\_contig-100\_1315.93779SRS013940\_contig\_number\_3615SRS014313\_contig\_number\_870SRS014412\_contig\_number\_19381SRS014534\_contig\_number\_985SRS014855\_contig\_number\_12106SRS015264\_contig\_number\_13873SRS015694\_contig\_number\_20931SRS015960\_contig\_number\_12924SRS016056\_contig\_number\_1947SRS016095\_contig\_number\_26220SRS016132\_contig\_number\_1326SRS017247\_contig\_number\_1040SRS017745\_contig\_number\_1735SRS018133\_contig\_number\_14397SRS018351\_contig\_number\_9451SRS018623\_contig\_number\_11998SRS018836\_contig\_number\_11789SRS018936\_contig\_number\_8607SRS018984\_contig\_number\_2115SRS019693\_contig\_number\_3344SRS019808\_contig\_number\_18521SRS019910\_contig\_number\_23492SRS020328\_contig\_number\_contig-100\_2277.204469SRS020508\_contig\_number\_488SRS021153\_contig\_number\_4062SRS021219\_contig\_number\_9135SRS022071\_contig\_number\_1379SRS022609\_contig\_number\_28966SRS023715\_contig\_number\_24369SRS024009\_contig\_number\_contig-100\_909.73030SRS024075\_contig\_number\_5123SRS024492\_contig\_number\_927SRS042284\_contig\_number\_4945SRS045004\_contig\_number\_2025SRS045645\_contig\_number\_6956SRS045713\_contig\_number\_6108SRS045826\_contig\_number\_3597SRS046369\_contig\_number\_9995SRS046717\_contig\_number\_2334SRS048060\_contig\_number\_10800SRS048164\_contig\_number\_8203SRS048981\_contig\_number\_1500SRS049402\_contig\_number\_1690SRS049896\_contig\_number\_10671SRS049995\_contig\_number\_13433SRS050026\_contig\_number\_2875SRS050422\_contig\_number\_9656SRS050752\_contig\_number\_19668SRS050801\_contig\_number\_1787SRS050941\_contig\_number\_20482SRS051031\_contig\_number\_30483SRS051610\_contig\_number\_8068SRS052078\_contig\_number\_525SRS053087\_contig\_number\_2468SRS053335\_contig\_number\_9378SRS053398\_contig\_number\_16667SRS054956\_contig\_number\_9552SRS056519\_contig\_number\_5249SRS056537\_contig\_number\_55945SRS058723\_contig\_number\_2113SRS063190\_contig\_number\_1258SRS063370\_contig\_number\_1682SRS063928\_contig\_number\_140SRS064645\_contig\_number\_252SRS065176\_contig\_number\_1283SRS075021\_contig\_number\_4313SRS075716\_contig\_number\_312SRS075821\_contig\_number\_13551SRS075963\_contig\_number\_contig-100\_1532.1532SRS076756\_contig\_number\_13585SRS076804\_contig\_number\_12407SRS076876\_contig\_number\_183SRS076929\_contig\_number\_20864SRS077024\_contig\_number\_3215SRS077194\_contig\_number\_13926SRS077231\_contig\_number\_10758SRS077335\_contig\_number\_2857SRS077552\_contig\_number\_1084SRS077730\_contig\_number\_10603SRS077849\_contig\_number\_5535SRS078176\_contig\_number\_contig-100\_1474.131352SRS098073\_contig\_number\_13031SRS098644\_contig\_number\_13796SRS098717\_contig\_number\_18996SRS101376\_contig\_number\_2576SRS103987\_contig\_number\_28257SRS1041031\_contig\_number\_24614SRS1041032\_contig\_number\_10133SRS1041033\_contig\_number\_23568SRS1041036\_contig\_number\_4427SRS1041112\_contig\_number\_1369SRS1041118\_contig\_number\_15426SRS1041133\_contig\_number\_14987SRS1041138\_contig\_number\_2114SRS1041142\_contig\_number\_1054SRS1041144\_contig\_number\_1081SRS1041147\_contig\_number\_1940SRS1041157\_contig\_number\_contig-100\_468.34839SRS1041164\_contig\_number\_1711SRS104327\_contig\_number\_155SRS1054691\_contig\_number\_11478SRS1055022\_contig\_number\_15220SRS1055038\_contig\_number\_210SRS1055049\_contig\_number\_10956SRS1055067\_contig\_number\_8786SRS1055069\_contig\_number\_232SRS1055076\_contig\_number\_5885SRS140513\_contig\_number\_9928SRS142503\_contig\_number\_15060SRS142542\_contig\_number\_5311SRS143085\_contig\_number\_1009SRS143181\_contig\_number\_10524SRS143342\_contig\_number\_1149SRS143372\_contig\_number\_8321SRS143466\_contig\_number\_29619SRS143523\_contig\_number\_3260SRS143895\_contig\_number\_39652SRS144135\_contig\_number\_29875SRS144714\_contig\_number\_24698SRS145308\_contig\_number\_2436SRS146764\_contig\_number\_contig-100\_2727.193981SRS146813\_contig\_number\_5362SRS147557\_contig\_number\_30897SRS147614\_contig\_number\_contig-100\_2031.54672SRS147919\_contig\_number\_7086SRS148091\_contig\_number\_9815SRS148721\_contig\_number\_32606SRS148970\_contig\_number\_1465SRS149244\_contig\_number\_315SRS149784\_contig\_number\_14399SRS149879\_contig\_number\_9480SRS150029\_contig\_number\_7338SRS893172\_contig\_number\_4983SRS893187\_contig\_number\_9826SRS893231\_contig\_number\_1292SRS893279\_contig\_number\_5051SRS893300\_contig\_number\_7153SRS893369\_contig\_number\_4159SRS893380\_contig\_number\_613SRS893382\_contig\_number\_2197SRS971275\_contig\_number\_22907


SRS016989\_contig\_number\_1049
204516
species
1

1
457391
species

SRS098655\_contig\_number\_8356


SRS046502\_contig\_number\_9534
1
28113
species


SRS104084\_contig\_number\_3654
1
2302926
species


SRS019286\_contig\_number\_980
1
species
2292913

4
species
47678

SRS075984\_contig\_number\_767SRS077294\_contig\_number\_69SRS144362\_contig\_number\_28101SRS147977\_contig\_number\_4054

species
2292950
5

SRS017307\_contig\_number\_27838SRS054059\_contig\_number\_1450SRS077086\_contig\_number\_6158SRS1041091\_contig\_number\_20163SRS1041134\_contig\_number\_2841

species
2292920
1

SRS098061\_contig\_number\_3143

2
species
162156

SRS077127\_contig\_number\_1877SRS893230\_contig\_number\_2340

2292944
species
1

SRS049900\_contig\_number\_14424


SRS011134\_contig\_number\_49760SRS012273\_contig\_number\_3576SRS013158\_contig\_number\_5169SRS013476\_contig\_number\_39139SRS014235\_contig\_number\_48462SRS014287\_contig\_number\_contig-100\_6409.83017SRS014613\_contig\_number\_1251SRS014948\_contig\_number\_23625SRS015369\_contig\_number\_5486SRS015578\_contig\_number\_7459SRS015663\_contig\_number\_36819SRS016018\_contig\_number\_11217SRS016753\_contig\_number\_8466SRS016897\_contig\_number\_561SRS017103\_contig\_number\_7083SRS017433\_contig\_number\_9998SRS018049\_contig\_number\_contig-100\_949.950SRS018888\_contig\_number\_5908SRS019397\_contig\_number\_9988SRS019968\_contig\_number\_17609SRS020394\_contig\_number\_14931SRS021484\_contig\_number\_28194SRS043001\_contig\_number\_1893SRS044535\_contig\_number\_10731SRS045244\_contig\_number\_contig-100\_6199.44923SRS050925\_contig\_number\_contig-100\_2000.173991SRS053649\_contig\_number\_1678SRS062427\_contig\_number\_contig-100\_1328.62560SRS063518\_contig\_number\_31379SRS064232\_contig\_number\_3539SRS064276\_contig\_number\_19806SRS064557\_contig\_number\_8689SRS064757\_contig\_number\_24465SRS065397\_contig\_number\_10274SRS074670\_contig\_number\_2764SRS076976\_contig\_number\_4016SRS077392\_contig\_number\_contig-100\_1546.212116SRS077502\_contig\_number\_1902SRS077589\_contig\_number\_1640SRS077641\_contig\_number\_21247SRS078242\_contig\_number\_29502SRS078260\_contig\_number\_contig-100\_1649.26767SRS078665\_contig\_number\_7598SRS098571\_contig\_number\_14602SRS1041037\_contig\_number\_13866SRS1041095\_contig\_number\_contig-100\_2194.36291SRS1041129\_contig\_number\_12580SRS1041140\_contig\_number\_contig-100\_8778.84756SRS1041145\_contig\_number\_4342SRS104311\_contig\_number\_40286SRS104400\_contig\_number\_13710SRS104975\_contig\_number\_653SRS140492\_contig\_number\_21680SRS142980\_contig\_number\_3014SRS143598\_contig\_number\_14423SRS143780\_contig\_number\_10180SRS143991\_contig\_number\_23133SRS144506\_contig\_number\_2261SRS144537\_contig\_number\_45893SRS145497\_contig\_number\_contig-100\_866.88374SRS146812\_contig\_number\_51507SRS147039\_contig\_number\_3984SRS147139\_contig\_number\_29928SRS148319\_contig\_number\_12485SRS149075\_contig\_number\_17164SRS149325\_contig\_number\_contig-100\_1238.100688SRS893187\_contig\_number\_contig-100\_2885.100684SRS893378\_contig\_number\_19975SRS971276\_contig\_number\_29219
1946083
species
69

17
species
1946089

SRS014923\_contig\_number\_16789SRS015794\_contig\_number\_6032SRS018427\_contig\_number\_463SRS019068\_contig\_number\_18250SRS019496\_contig\_number\_2536SRS024075\_contig\_number\_contig-100\_8240.8240SRS024132\_contig\_number\_4409SRS024625\_contig\_number\_21317SRS054905\_contig\_number\_2178SRS101433\_contig\_number\_3066SRS1041139\_contig\_number\_3109SRS144297\_contig\_number\_12133SRS147766\_contig\_number\_7188SRS148424\_contig\_number\_1502SRS893270\_contig\_number\_9318SRS893366\_contig\_number\_10345SRS893383\_contig\_number\_736

13
species
46506

SRS014683\_contig\_number\_12369SRS017859\_contig\_number\_182SRS017859\_contig\_number\_1492SRS020233\_contig\_number\_10476SRS024663\_contig\_number\_10780SRS042966\_contig\_number\_20547SRS045739\_contig\_number\_contig-100\_2643.67714SRS049773\_contig\_number\_2928SRS062654\_contig\_number\_3447SRS1041116\_contig\_number\_6938SRS144603\_contig\_number\_17278SRS893292\_contig\_number\_2977SRS893358\_contig\_number\_4820

1
family
1853231

283168
genus
1


SRS023526\_contig\_number\_12545
1
28118
species

3
2005525
family

375288
genus
2

1
46503
species

SRS018575\_contig\_number\_986


SRS148817\_contig\_number\_3170
1
species
823

195950
genus
1

species
2293248
1

SRS016954\_contig\_number\_22385

1
family
171550

239759
genus
1


SRS058770\_contig\_number\_21943
species
1465754
1
